# Complement Activation: An Emerging Player in the Pathogenesis of Cardiovascular Disease

**DOI:** 10.6064/2012/402783

**Published:** 2012-12-16

**Authors:** Angela M. Carter

**Affiliations:** Division of Epidemiology, Leeds Institute of Genetics, Health and Therapeutics, Faculty of Medicine and Health and the Multidisciplinary Cardiovascular Research Centre, University of Leeds, Clarendon Way, Leeds LS2 9JT, UK

## Abstract

A wealth of evidence indicates a fundamental role for inflammation in the pathogenesis of cardiovascular disease (CVD), contributing to the development and progression of atherosclerotic lesion formation, plaque rupture, and thrombosis. An increasing body of evidence supports a functional role for complement activation in the pathogenesis of CVD through pleiotropic effects on endothelial and haematopoietic cell function and haemostasis. Prospective and case control studies have reported strong relationships between several complement components and cardiovascular outcomes, and *in vitro* studies and animal models support a functional effect. Complement activation, in particular, generation of C5a and C5b-9, influences many processes involved in the development and progression of atherosclerosis, including promotion of endothelial cell activation, leukocyte infiltration into the extracellular matrix, stimulation of cytokine release from vascular smooth muscle cells, and promotion of plaque rupture. Complement activation also influences thrombosis, involving components of the mannose-binding lectin pathway, and C5b-9 in particular, through activation of platelets, promotion of fibrin formation, and impairment of fibrinolysis. The participation of the complement system in inflammation and thrombosis is consistent with the physiological role of the complement system as a rapid effector system conferring protection following vessel injury. However, in the context of CVD, these same processes contribute to development of atherosclerosis, plaque rupture, and thrombosis.

## 1. Introduction

Cardiovascular disease (CVD) is a leading cause of morbidity and mortality worldwide. Major modifiable risk factors for cardiovascular disease include smoking, physical inactivity, poor diet, and obesity, factors which contribute to a proinflammatory state [1]. Inflammation is recognised to play fundamental role in the pathogenesis of CVD, contributing to the development and progression of atherosclerotic lesion formation, plaque rupture, and thrombosis [2]. The role of inflammatory processes is highlighted by studies demonstrating that elevated levels of inflammatory markers precede and predict the development of CVD and cardiovascular mortality [3–9]. The most widely studied inflammatory factor is C-reactive protein (CRP), which has consistently been shown to predict the development of CVD [10]. Whilst it is widely accepted that CRP is an important biomarker, it is also clear that CRP levels can be induced by a wide variety of stimuli, including acute and chronic infection, and are elevated in various disease processes associated with inflammation, indicating a lack of specificity [11, 12]. Whether CRP plays a functional role in CVD remains controversial [2, 12], although it has clearly been shown to be present in atherosclerotic plaques, colocalised with activated complement components [13, 14]. CRP is a member of the pentraxin family of pattern recognition molecules which recognises and binds to “foreign” molecules leading to activation of the classical complement cascade [15]; therefore, a potential mechanistic role for CRP in CVD may be mediated via complement activation. This paper provides an overview of the inflammatory processes underpinning development of CVD and the increasing body of evidence supporting a functional role for complement activation in the pathogenesis of CVD through pleiotropic effects on endothelial and haematopoietic cell function and haemostasis.

## 2. The Complement System

### 2.1. Activation of the Complement Cascade

**Figure 1:**
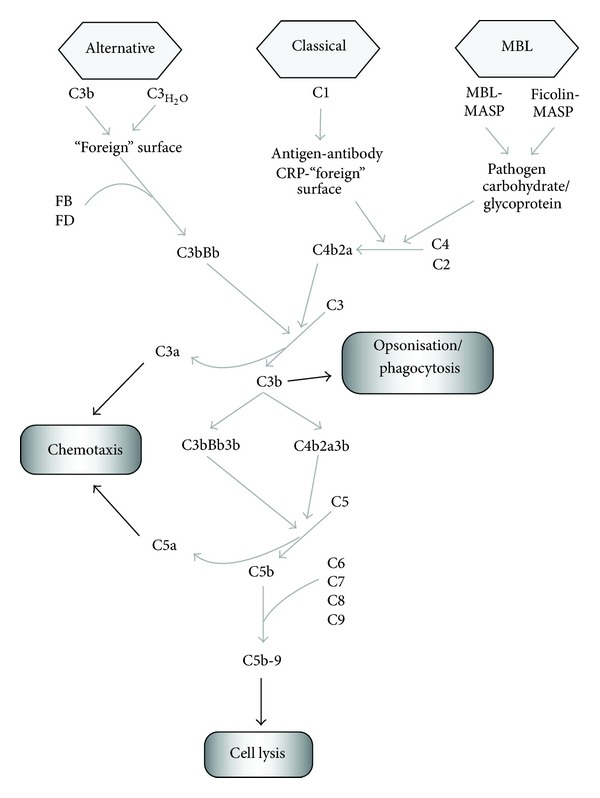
The 3 pathways of complement activation: classical, mannose-binding lectin (MBL), and alternative, which converge at formation of the C3 convertase complexes, C4b2a and C3bBb, which cleave C3, the main effector protein of the complement cascade, to C3a and C3b. C3b acts as an opsonin targeting C3b-bound “foreign” surfaces for phagocytosis. C3b also incorporates into the C3 convertase complexes to form C5 convertase complexes (C4b2a3b, C3bBb3B), which cleave C5 to C5a and C5b, with C5b subsequently participating in formation of the lytic C5b-9 complex. C3a and C5a are anaphylatoxins, promoting chemotaxis and mast cell degranulation.

The complement system plays a fundamental role in innate immunity in addition to enhancing adaptive immune responses and is therefore a primary line of defence against infection following injury [16]. Three different pathways of complement activation are known, the classical pathway, mannose-binding lectin pathway (MBL), and alternative pathways [17, 18], as shown in Figure 1. The classical pathway involves antigen/antibody or CRP/“foreign” molecule complexes interacting with C1 complex components (C1q, C1r, and C1s), leading to cleavage of C4 and C2 and formation of the classical C3 convertase, C4b2a [17]. The MBL pathway involves MBL or ficolin interactions with carbohydrate or glycoprotein moieties on pathogen surfaces and binding of MBL-associated serine proteases (MASP), also leading to cleavage of C4 and C2 and formation of C4b2a [19]. Whilst five MASP proteins are currently known (MASP 1–3, MAp19, MAP1), MASP-2 is required for activation of the MBL pathway, with MASP-1 acting to augment the action of MASP-2; the biological relevance of the other MASP proteins is largely unclear [20]. The alternative pathway is constitutively active as a result of low-level hydrolysis of the C3 thioester bond-generating C3_H_2_O_ [21]. Alternative pathway activation involves interaction of C3_H_2_O_ or C3b (generated by either the classical or MBL pathways) with factor B, which is cleaved by factor D to generate the alternative C3 convertase, C3_H_2_O_Bb or C3bBb [21]. Properdin enhances alternative cascade activation by stabilising the alternative C3 convertases, forming C3_H_2_O_BbP or C3bBbP, and anchors alternative C3 convertases to activating surfaces to enhance C3 cleavage [22].

The three activation pathways converge at the formation of the C3 convertases which cleave C3, the main effector protein of the complement cascade, to C3a and C3b (Figure 1). C3b acts as an opsonin targeting C3b-bound “foreign” surfaces for phagocytosis through interactions of C3b and C3b degradation products with complement receptors (CRs) 1 to 4. C3b also incorporates into the C3 convertase complexes to form C5 convertase complexes (C4b2a3b, C3bBb3b), which cleave C5 to C5a and C5b, with C5b subsequently participating in formation of the lytic C5b-9 complex (membrane attack complex, MAC; see Figure 1) [23]. C3a and C5a are anaphylatoxins which mediate their inflammatory activities, including chemotaxis and mast cell degranulation, via interaction with specific receptors C3aR and C5aR [24]. The complement system acts as a rapid first-line defence against pathogen invasion by opsonisation and lytic destruction [17, 25]. In addition, the complement system serves an important role in clearance of damaged “self” cells, by targeting apoptotic and necrotic cells for complement-mediated phagocytosis [26, 27].

The constitutive activation of the alternative pathway via generation of C3_H_2_O_ acts as a surveillance mechanism, enabling rapid responses to invading pathogens or damaged “self” cells. In addition, activation of the alternative pathway by C3b generated by classical or MBL pathways is an essential step in complement activation, forming an amplification loop for complement activation [28]. Indeed, it has been demonstrated that more than 80% of the C5a and C5b-9 generated by activation of the classical or MBL pathways arises via alternative pathway amplification [29, 30]. However, the amplification of complement activation via the alternative pathway has the potential to lead to devastating damage to “self” cells through massive generation of C3a, C5a, and C5b-9. Protection of “self” cells necessitates a complex network of regulatory molecules, both soluble (factor H, factor H-related protein-1, factor I, C1-inhibitor, C4 binding protein, vitronectin, clusterin, and carboxypeptidase N) and membrane bound (CR1, CD46, CD55, and CD59) [31, 32].

### 2.2. Regulation of Complement Activation

In the fluid phase, factor I inhibits complement by cleaving C3b to iC3b and cleaving C4b [33]; however, factor I is only active against these substrates in complex with cofactors: factor H or C4 binding protein in the fluid phase; CR1 and CD46 on membranes [34]. Factor H inhibits complement through its cofactor activity for factor I [33, 35] and is also the major fluid-phase inhibitor of the alternative C3 convertase by directly promoting decay of the alternative C3 convertase complex [36]. C4 binding protein (C4BP) is the major soluble inhibitor of the classical C3 convertase, preventing the formation of C4b2a and accelerating its decay [37, 38]. C4BP also acts as a cofactor for the inactivation of C4b by factor I [39] and is a cofactor for factor I-mediated cleavage of C3b thereby contributing to inhibition of the alternative pathway [40]. CD46, also known as membrane cofactor protein (MCP), acts as a cofactor for factor I in the degradation of C3b and C4b deposited on cell surfaces therefore inhibiting convertase formation [41, 42]. CR1 (CD35) acts as a membrane receptor for C3b and C4b, preventing formation and promoting decay of classical and alternative C3 and C5 convertase complexes [43, 44] and acts as a cofactor for FI-mediated cleavage of C3b and C4b [45, 46].

CD55, also known as decay accelerating factor (DAF) acts at the level of the alternative and classical C3 and C5 convertases. It functions both by preventing the formation of new C3 convertase complexes and by accelerating the decay of already formed convertases [47, 48]. C1 inhibitor inhibits classical pathway activation by inactivation of C1r and C1s components of the C1 complex [49, 50] and inactivates the MBL pathway via inhibition of MASP-2 [51]. Carboxypeptidase N is the major soluble inhibitor of C3a and C5a anaphylatoxins, acting by cleaving the terminal arginine residues to form C3adesArg and C5adesArg [52, 53] to significantly decrease their chemotactic activities.

Clusterin is a soluble inhibitor of C5b-9 complex formation, which binds to C7, C8*β*, and C9 to inhibit membrane insertion of C5b-7 and formation of membrane-bound C5b-9 by promoting formation of a soluble C5b-9 complex (SC5b-9) [54, 55]. Vitronectin acts in a similar fashion to clusterin by promoting formation of soluble C5-9 [56], a process involving interactions with the C5b and C8 components [57]. Factor H-related protein 1 (CFHR-1) is a complement regulator with structural similarity to factor H but lacking cofactor activity towards factor I and decay accelerating function towards alternative C3 convertase [58]. Recently, CFHR-1 has been demonstrated to inhibit alternative C5 convertase activity, and it also inhibits formation and membrane insertion of the C5b-9 complex [59]. CD59, also known as protectin, protects host cells from C5b-9 [60] by binding to the C8*α* subunit [61] and C9 [62] during complex formation to inhibit formation of the pore-forming C5b-9 by preventing incorporation of multiple C9 molecules [63].

### 2.3. Complement Deficiency and Disease

Consistent with the physiological role of the complement system, deficiencies (inherited or acquired) and defects in components of the complement cascade can result in increased susceptibility to infection, including meningitis, or increased susceptibility to complement-mediated tissue damage. Deficiencies in the classical pathway components also give rise to increased susceptibility to autoimmune disease, including systemic lupus erythematosus (SLE), thought to arise in part due to defective clearance of apoptotic cells [64]. Deficiency of C1 inhibitor is associated with the development of potentially life-threatening angioedema [65]. Deficiency of C3 and C4 is associated with glomerulonephritis arising due to the glomerula deposition of immune complexes [66]. Deficiency of factor H or factor I leads to unregulated complement activation resulting in acquired deficiency of C3 and C5 through consumption [66]. Deficiency or defects in factor H, factor I, CD46, and factor B are associated with development of atypical haemolytic uremic syndrome (aHUS), which is characterised by complement-mediated haemolysis, thrombosis, and renal damage [67, 68]. Deficiency in CD55 and CD59 is associated with paroxysmal nocturnal haemoglobinuria (PNH), which is associated with complement-mediated haemolysis and thrombosis [69]. Whilst the majority of the pathological consequences of complement dysregulation would be anticipated based on the function of complement, the accelerated development of atherosclerosis and increased susceptibility to thrombosis associated with defects in alternative complement regulation point to a potential pathophysiological role for complement in cardiovascular disease [70–72]. A wealth of evidence from clinical studies, animal models, and *in vitro* functional analyses now clearly supports a direct contribution of complement activation in the development of atherothrombotic cardiovascular disease, as outlined later.

## 3. The Complement System and Cardiovascular Disease

A number of prospective and case control studies have reported strong relationships between several complement components and cardiovascular outcomes. Studies have shown that C3 predicts future cardiovascular events in men and women [3, 9, 73, 74], and case control studies have shown that C3 is elevated in patients with CVD, independent of conventional cardiovascular risk factors including CRP [75–77]. Plasma C3 and C5a predict increased intima-media thickness in patients with systemic lupus erythematosus (SLE) [52], and C5a predicts future cardiovascular events in patients with peripheral arterial disease [78], suggesting that complement activation contributes to progression of atherosclerosis and acute thrombotic events [79]. Several studies have demonstrated that complement activity is upregulated during the acute phase of both MI [80–82] and ischaemic stroke [83], with complement activation contributing to ischemia/reperfusion injury [84] and influencing final infarct size [85]. Elevated levels of C3 at the time of an acute ischaemic event have also been associated with worse outcome [86], and elevated C3 predicts restenosis following carotid endarterectomy [87]. Further support for a functional role of complement activation in CVD comes from recent proteomics studies, showing differential expression of complement components C1, C3, C4, C5a, and factor B in plasma from patients with coronary artery stenosis (>50%) compared with subjects with normal arteries (no stenosis ≥ 25%) [88]. Components of the classical, alternative and common pathways of complement activation were also identified at sites of human coronary thrombosis, in addition to deposition of activated complement components C3d and C5b-9 [89]. Furthermore, the presence of C9 in myocardial tissue from autopsy has been demonstrated to be effective in detecting early MI [90, 91]. Taken together, these studies support a role for complement activation in the pathogenesis of atherosclerosis and thrombosis. 

### 3.1. Complement and Atherosclerosis

Atherosclerosis bears all the hallmarks of a chronic inflammatory disease, with the cellular component of early lesions comprising inflammatory monocyte-derived macrophages, T lymphocytes (predominantly Th1), and mast cells [92–94]. Under normal physiological conditions, the endothelium maintains vascular tone, inhibits cell adhesion, and suppresses activation of the coagulation cascade through the secretion of a variety of bioactive molecules including nitric oxide (NO), prostacyclin, and endothelin I, thereby regulating blood flow and maintaining vascular patency [95]. Endothelial cell activation represents the initiating process in the development of atherosclerosis, mediated by a variety of factors including reactive oxygen species, leading to a proinflammatory, provasoconstrictive, and prothrombotic endothelial cell phenotype [95]. The proinflammatory phenotype is characterised by increased expression of a variety of cellular adhesion molecules, including E-selectin, P-selectin, vascular cell adhesion molecule-1 (VCAM-1), and intercellular adhesion molecule 1 (ICAM-1). These adhesion molecules facilitate binding of inflammatory cells to the activated endothelium and migration into the arterial intima, initiating an inflammatory response within the arterial wall [2]. Within the intima, monocytes are transformed into macrophages which internalise oxidised low-density lipoprotein (oxLDL) and promote macrophage conversion to foam cells and formation of the fatty streak [92]. Foam cells secrete a variety of inflammatory cytokines and chemokines, promoting increased expression of endothelial cell adhesion molecules and ongoing leukocyte accumulation in the arterial intima, thereby enhancing the local inflammatory response within the arterial wall [2, 96]. Continuing production of inflammatory mediators leads to intimal migration and proliferation of vascular smooth muscle cells (VSMC), which synthesise and secrete collagen, leading to expansion of the extracellular matrix and formation of a complex atherosclerotic lesion overlayed by a fibrous cap [92]. Secretion of cytokines by various cellular components of the plaque promotes macrophage, endothelial cell, and VSMC expression of tissue factor (TF), a potent activator of blood coagulation [2, 96]. The internal plaque environment therefore becomes highly prothrombotic and is shielded from the blood by the fibrous cap. Thinning of the plaque cap results in increased susceptibility to plaque rupture, particularly at the shoulder region of the cap, exposing subendothelial plaque components rich in TF to circulating blood, leading to thrombus formation [2].

Increased C3 deposition within the intima of human atherosclerotic lesions compared with normal vessel intima, in the absence of complement deficiencies or defects, provided support for the suggestion that complement may play a direct functional role in atherosclerosis [97–104]. The presence of C5b-9 within atherosclerotic plaques, from the earliest to advanced lesions, indicated that full complement activation occurs within the plaque [102, 105, 106]. Increased expression of complement mRNAs in atherosclerotic plaques, compared with normal tissue, demonstrated active synthesis of complement by the cellular components of the plaque [107]. Regulators of complement activation, both soluble and membrane bound, have also been identified in atherosclerotic plaques, suggesting localised regulation of complement activation [106, 108, 109]. Together, these observations strongly supported localised generation of complement components, complement activation, and complement regulation; however, the consistent identification of C5b-9 within plaques indicates inefficient complement inhibition and suggests an active contribution to intravascular inflammation and atherosclerosis. 

Complement activation *in vitro* influences many processes involved in the development and progression of atherosclerosis, including promotion of endothelial cell activation, monocyte infiltration into the extracellular matrix, and stimulation of cytokine release from VSMCs [98, 110]. C5a and C5b-9 interact with endothelial cells giving rise to upregulation of cellular adhesion molecules including P-selectin, E-selectin, ICAM-1, and VCAM-1, therefore contributing to endothelial cell activation and promotion of leukocyte infiltration into the vessel wall [110]. Lipid components isolated from atherosclerotic lesions have long been known to activate the alternative complement cascade [42, 111]. Enzymatically modified LDL (E-LDL) has been identified as the lipid component responsible for alternative complement activation [112], and recent studies indicate that the extent of complement activation is dependent upon the concentration of E-LDL and is influenced by CRP [113]. Specifically, at lower concentrations of E-LDL complement activation is dependent upon binding of CRP and is limited to cleavage of C3 to liberate C3 and C3b, but activation to the level of C5 cleavage is limited by CRP/factor H interactions [13], whereas at higher concentration of E-LDL, complement activation is independent of CRP and leads to full complement activation [113]. More recently, E-LDL has also been shown to activate the classical complement pathway via interaction of C1q with E-LDL, in a CRP independent manner [114]. These studies therefore support the *in vivo* relevance and linking complement activation with the earliest stages of atheroma formation. C3a and C5a are potent mediators of inflammation and chemotaxis and serve to recruit monocytes and T-lymphocytes and promote leukocyte synthesis of IL6, IL1*β*, and TNF*α* to enhance inflammatory processes [2, 98]. C5b-9 generated on human VSMC leads to the secretion of monocyte chemoattractant protein-1 (MCP-1), indicating additional mechanisms by which C5b-9 could promote accumulation of monocytes within the arterial intima [115]. C5a has also been shown to upregulate macrophage expression of matrix metalloproteinase-1 (MMP-1) and MMP-9, suggesting a role for complement activation in extracellular matrix degradation and plaque destabilisation [116]. C3a and C5a also induce mast cell degranulation [117] and promote mast cell synthesis of PAI-1, leading to a prothrombotic phenotype [118]. Taken together, these data indicate the potential for complement activation to contribute to all stages of atheroma formation, including endothelial cell dysfunction, recruitment of inflammatory cells and VSMCs into the arterial intima, and increased susceptibility to plaque rupture. The identification of complement cleavage products within plaques, colocalisation of C5b-9 with plaque macrophages and cell debris, and expression of CR1 and CR3 by plaque macrophages lends support for a role of ongoing complement activation in the pathogenesis of atherosclerosis [99–104, 119]. Enhanced activation of complement in ruptured compared with stable plaques also suggests concurrent complement activation and thrombosis [99]. 

### 3.2. Complement and Thrombosis

The exposure of subendothelial collagen and vWF to circulating platelets upon plaque rupture initiates thrombus formation by promoting platelet adhesion, activation, and aggregation. Exposure of lesion-associated TF to plasma factor VII results in activation of the coagulation cascade and thrombin generation on the platelet surface. Thrombin promotes further platelet activation, cleavage of fibrinogen to form fibrin, and activation of factor XIII, leading to formation of a platelet-rich cross-linked fibrin clot able to withstand mechanical pressure and proteolytic degradation [120]. Fibrin formation is counteracted by the fibrinolytic system, in particular involving tissue-type plasminogen activator (tPA), which cleaves plasminogen to form the serine protease plasmin, which degrades fibrin to limit the extent of thrombus formation [121, 122]. Fibrinolytic inhibitors, including plasminogen activator inhibitor-1 (PAI-1) and thrombin-activatable fibrinolysis inhibitor (TAFI), ensure that the fibrinolytic process is regulated to prevent inappropriate dissolution of the thrombus. The formation of *ex vivo* fibrin clots with denser structures, decreased porosity, and prolonged fibrinolysis times is observed in patients with CVD [123]. Similar clot properties have been demonstrated in healthy individuals at increased risk for CVD [124–126], suggesting alterations in clot structure and susceptibility to fibrinolysis influence development of CVD. Thrombin generation is counteracted by anticoagulant factors, in particular endothelial cell thrombomodulin which forms a complex with thrombin leading to activation of protein C. Activated protein C inactivates factors Va and VIIIa to dampen down the positive amplification loop of thrombin generation [120, 127]. Consequently, the extent of thrombus formation upon plaque rupture and the resulting clinical sequelae are influenced by factors regulating the balance between prothrombotic, anticoagulant, and pro- and antifibrinolytic stimuli [2]. 

There is mounting evidence from *in vivo* and *in vitro* studies to support a functional role for complement activation in the thrombotic component of acute CVD, including the cellular and fluid phases of haemostasis [72]. Activation of complement influences endothelial function via a number of mechanisms. Activation of complement and generation of C5b-9 induces endothelial cell TF expression and secretion of vWF to induce a prothrombotic endothelial cell phenotype with the potential to activate the coagulation cascade, leading to thrombin generation, and promote platelet activation and adhesion [110, 128]. C5b-9 also upregulates endothelial expression of CD55 [129], suggesting a potential negative feedback loop whereby terminal complement activation leads to downregulation of C3 and C5 convertase activity to limit subsequent generation of C5a and C5b-9. Thrombin also upregulates endothelial cell CD55 expression, suggesting that interactions between complement activation and activation of coagulation on the endothelial cell surface are important for dampening complement-mediated proinflammatory and procoagulant processes to protect from excessive formation of C5b-9 and to limit thrombin generation and thrombus formation on intact endothelium [130]. 

Numerous interactions between complement components and platelets have been described which support a role of complement activation in thrombosis. Activated platelets have been demonstrated to have an intrinsic ability to activate both classical and alternative complement pathways [131]. Activation of platelets by shear stress leads to platelet-mediated activation of the classical complement pathway involving C1q interactions with gC1qR [131–133]. Activation of platelets with potent agonists, including thrombin and arachidonic acid, promotes activation of the alternative complement pathway mediated via binding of C3b to P-selectin [131, 134]. C1q interaction with platelets has also been shown to induce expression of P-selectin on the platelet surface to potentially enhance complement activation via the alternative pathway [135]. C1q interaction with platelets also reduces collagen-mediated platelet activation via GPVI and platelet-neutrophil aggregate formation, a process dependent upon P-selectin interaction with PSGL-1 [77], suggesting a negative feedback on collagen-mediated platelet adhesion to potentially favour enhanced complement activation. Interestingly, the complement components which form the C5b-9 complexes are secreted by activated platelets, suggesting that platelet activation may promote the localised formation of C5b-9 [136]. Complement activation occurring on the platelet surface leads to generation of C3a, C5a, and C5b-9, which have been shown to induce platelet activation, *α*-granule release, and aggregation [74, 75]. Therefore, complement activation on platelets can lead to a positive feedback loop for activation of platelets and complement. C5b-9 also induces platelet microparticle formation from activated platelets, and induces exposure of binding sites for FVIIIa and FVa on platelet and microparticle membranes leading to tenase and prothrombinase complex formation, thereby promoting thrombin generation on the platelet surface [137–140]. C5b-9 therefore enhances platelet activation and aggregation and promotes thrombin generation on the platelet surface and fibrin formation, suggesting that complement activation may be important in consolidation of a forming thrombus. 

The complement and coagulation cascades act in concert as rapid effector systems for preventing blood loss and protecting against infection following vascular injury, and interactions between complement and coagulation cascade components are increasingly being described [72]. Given the colocalisation and coordinated activation of these two cascades and the fact that they derive from common ancestral genes [141], it is suggested that these interactions are likely to be of physiological and pathophysiological relevance. Activation of the alternative complement cascade has been shown to give rise to fibrin clots with a denser structure that lyse more slowly, although the complement component(s) mediating these effects were not initially characterised [142]. In recent studies, we have identified C3 as a novel clot component in perfused solubilised plasma clots, and functional analyses indicated that C3 bound to fibrin with high affinity [86]. C3 impaired fibrinolysis in a concentration dependent manner in a purified system in the absence of C3 convertase complexes and in plasma-based systems, suggesting a direct effect of C3 on fibrinolysis even in the presence of physiologically relevant fibrinolysis and complement inhibitors [86]. In clinical studies, we have demonstrated that elevated C3 is independently associated with prolonged fibrinolysis times after accounting for haemostatic determinants in healthy individuals [143] and individuals with diabetes [144, 145], supporting a functional relationship between elevated C3 and prolonged fibrinolysis. Numerous other studies have demonstrated functional interactions between complement components and fibrin formation. In the fluid phase, MBL-associated serine protease-1 (MASP1) cleaves fibrinogen to fibrin and activates FXIII to generate cross-linked fibrin, although with greatly reduced catalytic efficiency compared with thrombin [92]. MASP1 also activates prothrombin, although with greatly reduced efficiency compared with FXa [89]. MASP-MBL complexes or L-ficolin-MBL complexes incubated with plasma or purified fibrinogen and FXIII also generated cross-linked fibrin [146], although MASP1-mediated fibrin formation in plasma was demonstrated to be secondary to MASP-1 cleavage of prothrombin [147]. MASP1 has also been shown to cause prolongation of fibrinolysis and to directly cleave TAFI, again with greatly reduced catalytic activity compared with thrombin, suggesting that prolonged fibrinolysis may be dependent upon activation of TAFI [147]. Similar to MASP1, MASP2 also cleaves prothrombin to generate thrombin and initiate fibrin formation [148], suggesting coordinated activation of the MBL pathway and initiation of thrombin generation. Serine proteases of the coagulation cascade are also capable of activating complement, with thrombin and activated coagulation factors IXa, Xa, and XIa capable of directly cleaving C3 and C5 to generate functional C3a and C5a [149–151]. FXIIa has also been demonstrated to initiate classical pathway activation via cleavage of C1r [152]. Complement and haemostatic mechanisms also interact at the inhibitor level, for instance, C1-inhibitor inhibits complement factors C1r, C1s, MASP1, and MASP2, as well as coagulation factors XIIa and XIa [153]. TAFI not only inhibits fibrinolysis by cleaving terminal lysine residues from fibrin, thereby preventing plasminogen binding and plasmin generation [154], but TAFI also cleaves terminal arginine residues from C3a and C5a to downregulate their proinflammatory activities [155]. Cross-talk between complement and coagulation cascades therefore occurs at multiple levels and appears to be important in coordination to haemostatic and immune responses.

*In vitro* studies and clinical observational studies indicate strong links between complement and CVD; however, they cannot directly indicate a causal relationship. Consequently, studies employing animal models have been carried out to further explore the functional effects of complement in CVD, in particular by evaluating the effects of deficiency of one or more complement components on the development of atherosclerosis and thrombosis, detailed later.

## 4. Animal Models of Complement and Cardiovascular Disease

Studies in animals support functional relationships between complement activation and the pathogenesis of atherosclerosis and thrombosis. In rabbits fed a high-fat diet, C5b-9 expression was detected in the intima preceding monocyte infiltration and foam cell formation [156], and C5b-9 also impaired endothelium-dependent vasorelaxation [157], indicating a role for complement activation in endothelial dysfunction and the early stages of atheroma formation. Also in high-fat fed rabbits, the extent of atherosclerosis was greatly reduced in C6-deficient compared with wild-type (WT) animals [158], supporting a functional role for the C5b-9 complex in the development and progression of atherosclerosis.

A number of studies have evaluated atherosclerosis risk associated with complement activation in mice on different atherosclerosis-prone backgrounds, including apolipoprotein E knockout (apoE^−/−^), LDL receptor knockout, LDLR^−/−^, and combined apoE^−/−^ LDLR^−/−^ mice. Inhibition of classical complement activation by C1 inhibitor in apoE^−/−^  mice resulted in decreased neointima formation and a reduction in plaque C3 deposition [159], suggesting that classical pathway activation and intraplaque deposition of C3 are associated with atheroma formation. In studies of C3-deficient mice (C3^−/−^) on an LDLR^−/−^ [160] and combined apoE^−/−^ LDLR^−/−^ [161] background, more extensive atheroma formation was observed in C3^−/−^ compared with complement sufficient mice during the early stages of atheroma formation [160, 161] but not after more prolonged periods [161]. The results of these studies are inconsistent with the data relating to C1 inhibition and with the hypothesis that complement activation contributes to the pathogenesis of CVD, since C3^−/−^ mice on an proatherogenic background would be expected to be protected from atherosclerosis. Further insight may be shed on this inconsistency by a recent study by Huber-Lang et al. who demonstrated that C3^−/−^ mice generated C5a even in the absence of C3 [150]. Generation of C5a was reduced in the presence of antithrombin III, and thrombin was capable of generating C5a *in vitro,* indicating a direct role for thrombin in activation of C5 [150]. The observation that C5a is generated in the absence of C3 in C3^−/−^ mice may explain the lack of association between C3^−/−^ and C3-sufficient mice in the development of atherosclerosis given the studies indicating a role for C5a and C5b-9 in particular in the processes underpinning the development and progression of atherosclerosis. Deficiency of C5 in apoE^−/−^ mice was not associated with increased aortic lesion area [162], which appears at variance with the previous hypothesis. However, deficiency of CD55, the cellular inhibitor which promotes dissociation of C3 convertase complexes and reduced C5 convertase formation leading to decreased generation of C3a, C5a, and C5b-9, resulted in increased neointimal thickening formation 14 days after wire-induced endothelial injury, which was associated with increased accumulation of macrophages and neutrophils [163]. The effects on neointimal thickening were reversed in CD55^−/−^ mice doubly deficient in either C3aR or C5aR, and inflammatory cell infiltration and cellular proliferation were reduced in C3aR^−/−^ and C5aR^−/−^ mice. Together, these data suggest that CD55 plays an essential role in downregulating generation of C3a and C5a [163] and indicates a role for C3a and C5a in atherosclerosis and restenosis through interactions with their respective receptors. A role for CD55 in atherosclerosis is also supported by the study of Leung et al. who demonstrated that CD55^−/−^ mice on an LDLR^−/−^ background developed larger and more complex lesions, characterised by increased VSMC and collagen content, and increased expression of C5b-9 compared with CD55 sufficient mice [164]. Consistent with a role for C5a in atherosclerosis, inhibition of C5aR with antagonist or blocking monoclonal antibody for 7 days after wire-induced endothelial injury in apoE^−/−^ mice reduced neointimal thickening, monocyte/macrophage and neutrophil infiltration, and expression of VCAM-1 and PAI-1 [165]. Furthermore, deficiency of CD59, the primary inhibitor of C5b-9 formation, in apoE^−/−^ mice resulted in enhanced C5b-9 formation leading to endothelial dysfunction, accelerated atherosclerosis, formation of vulnerable plaques, and premature death [166]. The effects of CD59 deficiency were attenuated by administration of a blocking antibody to C5 in CD59^−/−^ apoE^−/−^ mice and targeted overexpression of human CD59 in endothelial and haemopoietic cells in apoE^−/−^ mice resulted in resistance to the development of atherosclerosis, supporting a functional role for C5b-9 in the development and progression of atherosclerosis, plaque rupture, and thrombosis [110]. C6 deficiency in apoE^−/−^ mice, resulting in an inability to form C5b-9 complexes, resulted in more than a 50% decrease in atherosclerotic plaque area compared with control mice, therefore strongly supporting a functional role for C5-9 in progression of atherosclerosis [167].

Further support for the *in vivo* relevance of complement activation in thrombosis is derived from studies of MBL^−/−^ deficient and MASP1^−/−^ MASP3^−/−^ doubly deficient mice in which prolonged bleeding times and impaired thrombosis were observed following FeCl_3_-induced injury [168]. Additional support for a role of complement in thrombosis derives from studies in C3^−/−^ mice in which prolonged bleeding times, defective PAR4 peptide-induced platelet aggregation, and reduced thrombus deposition following *in vivo* laser-induced endothelial injury were observed, supporting a direct role for C3 in platelet activation and thrombosis [169]. Interestingly, PAR4 is the principal receptor mediating thrombin activation in murine platelets, although it is also readily activated by plasmin [170]. Therefore, impaired PAR4 peptide-specific platelet aggregation and reduced thrombus formation seems at odds with a predominant role for C5b-9 in platelet activation and the proposal that thrombin generation is upregulated in C3^−/−^ mice and bypasses C5 convertase formation to enable generation of C5a and C5b-9. However, since C3b interactions with P-selectin on thrombin-activated platelets are important in promoting activation of complement and formation of C5b-9 on the platelet surface leading to a positive feedback loop of complement and platelet activation [134], impaired PAR4-dependent platelet activation in C3^−/−^ mice may suggest an important role for C3b in propagating complement and platelet activation. 

Further *in vivo* support for a role of complement in acute arterial thrombosis derives from animal models of transient ischaemic stroke induced by short-term middle cerebral artery occlusion (MCAO). MCAO in C3^−/−^ mice resulted in decreased infarct size and improved neurological deficit compared with WT controls [171, 172], and this was associated with decreased P-selectin expression and inflammatory cell infiltration and decreased thrombus formation [172]. In support of a functional role for C3 in thrombosis, in a baboon model of sepsis-induced thrombosis, the C3 inhibitor compstatin, which binds to C3 and inhibits cleavage of  C3 by C3 convertase complexes, resulted in reduced TF and PAI-1 expression and decreased microvascular thrombosis [173]. MCAO in MBL^−/−^ mice resulted in decreased infarct size and improved neurologic score [174], whereas in this study, no difference in infarct size or functional outcome was observed in C1q^−/−^ and C5^−/−^ mice [171]. The lack of effect of C5 deficiency is again at odds with the proposed role for C5b-9 in thrombosis; however, CD59 deficiency also results in increased infarct size and impaired neurologic function following MCAO [175], therefore suggesting that C5b-9 is indeed associated with increased arterial thrombosis. 

Although there are some inconsistencies in the results of individual animal models of atherothrombotic CVD, taken together, these studies support a functional role for complement activation in both atherosclerosis and thrombosis. Taken together with human autopsy studies and *in vitro* studies, current evidence supports functional roles for C5a and C5b-9 in particular in the pathogenesis of atherosclerosis and thrombosis, although contributions of other complement components appear to be important particularly in thrombosis.

## 5. Conclusions and Future Perspectives

Substantial evidence suggests that complement activation contributes to endothelial cell activation, leukocyte and VSMC migration, platelet adhesion, activation and aggregation, activation of coagulation, and impaired fibrinolysis. The participation of the complement system in these processes is entirely consistent with the physiological role of the complement system as a rapid effector system conferring protection against infection following injury. These same interactions are likely to be of pathological relevance within the arterial system by promoting the development and progression of atherosclerosis, plaque rupture, thrombosis, and arterial occlusion giving rise to acute atherothrombotic events. Components of the complement system therefore represent novel biomarkers for CVD and potential targets for the development of novel cardioprotective agents; indeed, studies targeting complement in CVD have already taken place. For instance, pexelizumab is a humanised monoclonal antibody which inhibits activation of C5, which was shown to be effective in reducing infarct size after occlusion of the left anterior descending coronary artery in pigs [176]. Promising results were also obtained from an early study in humans in which administration of pexelizumab in patients with MI prior to percutaneous coronary intervention (PCI) reduced 90-day mortality, although no effect on infarct size was observed [177]. Unfortunately, in the large multicentre APEX-AMI trial of ~6000 patients, no beneficial effects of pexelizumab on 30-day mortality following PCI were observed [178]. The lack of significant effect on infarct size and mortality in this large study may seem at odds with a role for C5 activation components in atherothrombosis. However, differences between the highly controlled experimental model of ischaemia/reperfusion in pigs in which pexelizumab was administered prior to ischaemia [176] cannot be readily translated into studies of post-MI patients. These studies therefore suggest that short-term inhibition of C5 activation may not be effective in improving outcomes following PCI in patients with MI; however, no inference regarding more long-term inhibition of C5 activation can be made. Further studies are clearly required to evaluate the potential efficacy of chronic inhibition of complement activation in the context of primary and/or secondary prevention of acute atherothrombotic events.
